# Artificial Intelligence and Machine Learning in Rheumatology and Systemic Inflammatory Diseases: From Pattern Recognition to Signal Analysis and Clinical Decision Support

**DOI:** 10.3390/jcm15176864

**Published:** 2026-09-04

**Authors:** Matteo Colina, Roberto Diversi

**Affiliations:** 1 Rheumatology Service, Section of Internal Medicine, Department of Medicine and Oncology, Ospedale Santa Maria della Scaletta, 40026 Imola, Italy; 2 Department of Electrical, Electronic and Information Engineering, University of Bologna, Viale del Risorgimento 2, 40236 Bologna, Italy; roberto.diversi@unibo.it

**Keywords:** artificial intelligence, machine learning, rheumatology, inflammatory bowel disease, deep learning, fourier transform, vasculitis, digital biomarkers, precision medicine, large language models, nosological discovery

## Abstract

Artificial intelligence (AI) and machine learning (ML) are transforming the landscape of rheumatological and systemic inflammatory disease management, offering unprecedented capacity to integrate complex, multidimensional data for diagnostic support, disease monitoring, and therapeutic decision-making. This comprehensive narrative review, based on a non-systematic literature search of PubMed/MEDLINE and Google Scholar combined with the authors’ clinical expertise, provides a clinically oriented synthesis of current and emerging AI applications across the full spectrum of immune-mediated inflammatory diseases—including rheumatoid arthritis, systemic lupus erythematosus, vasculitis, inflammatory bowel disease, psoriatic arthritis, systemic sclerosis, inflammatory myopathies, and sarcoidosis—with particular attention to applications that have demonstrated or are approaching clinical utility. We discuss deep learning-based image analysis, natural language processing of electronic health records, multi-omic biomarker discovery, and the application of Fourier transform-based signal processing to biological time series as a novel approach to continuous disease monitoring. Fourier transform methods—already foundational in MRI reconstruction, cardiac electrophysiology, and clinical neurophysiology—are here systematically extended to rheumatological and inflammatory disease signals, including accelerometry, electromyography, heart rate variability, and longitudinal biomarker time series. The phenomenon of large language model hallucination—particularly critical in rare inflammatory diseases—is addressed alongside retrieval-augmented generation as a mitigation strategy. We further argue that AI-driven methods do not merely improve the interpretation of clinical data, but fundamentally expand what is observable—with profound epistemological implications for clinical knowledge transmitted through generations of medical tradition. Ethical considerations and future directions toward precision inflammatory disease medicine are outlined.

## 1. Introduction

Immune-mediated inflammatory diseases (IMIDs)—encompassing rheumatoid arthritis (RA), systemic lupus erythematosus (SLE), ANCA-associated vasculitis (AAV), inflammatory bowel disease (IBD), psoriatic arthritis (PsA), systemic sclerosis (SSc), inflammatory myopathies, and numerous related conditions—share fundamental pathogenetic mechanisms involving dysregulated innate and adaptive immunity, but differ substantially in their clinical phenotypes, organ tropism, diagnostic criteria, and therapeutic targets. Despite remarkable advances in classification, targeted therapies, and biomarker discovery over the past two decades, the clinical management of IMIDs continues to face profound challenges.

Diagnosis is frequently delayed because many IMIDs share overlapping clinical and laboratory features, and their onset is often insidious—particularly in early or undifferentiated presentations where differential diagnosis can be challenging even for experienced clinicians. In axial spondyloarthritis, the global mean diagnostic delay exceeds 7.4 years from symptom onset [1]; in systemic sclerosis, Raynaud’s phenomenon may precede overt connective tissue disease by decades; in ANCA-associated vasculitis, non-specific constitutional symptoms frequently delay recognition by months to years. This diagnostic challenge is compounded by the dependence of most IMIDs on composite clinical indices that capture only a partial picture of underlying biological activity and are subject to significant inter-observer variability.

Artificial intelligence (AI) and machine learning (ML)—computational approaches that enable systems to learn patterns from data and improve performance without explicit programming—are emerging as transformative tools across this spectrum. These disciplines are particularly well-suited to IMID management: the diseases generate large volumes of structured and unstructured data (laboratory values, imaging findings, endoscopic reports, histopathology, clinical notes, patient-reported outcomes), rely heavily on pattern recognition for diagnosis, and face precisely the kind of high-dimensional complexity that ML algorithms are designed to handle.

This review provides a comprehensive, clinically oriented synthesis of current and emerging AI and ML applications across the major IMIDs, with particular attention to applications that have demonstrated or are approaching clinical utility. We cover diagnostic support, disease monitoring, relapse prediction, signal processing techniques including Fourier transform-based analysis, and the emerging role of large language models. We further argue that AI-driven methods do not merely improve interpretation of existing observations, but fundamentally expand the boundaries of what is observable in inflammatory disease—with epistemological implications that extend to established clinical knowledge itself.

## 2. Materials and Methods

### Literature Search and Selection Strategy

**Figure 1:**
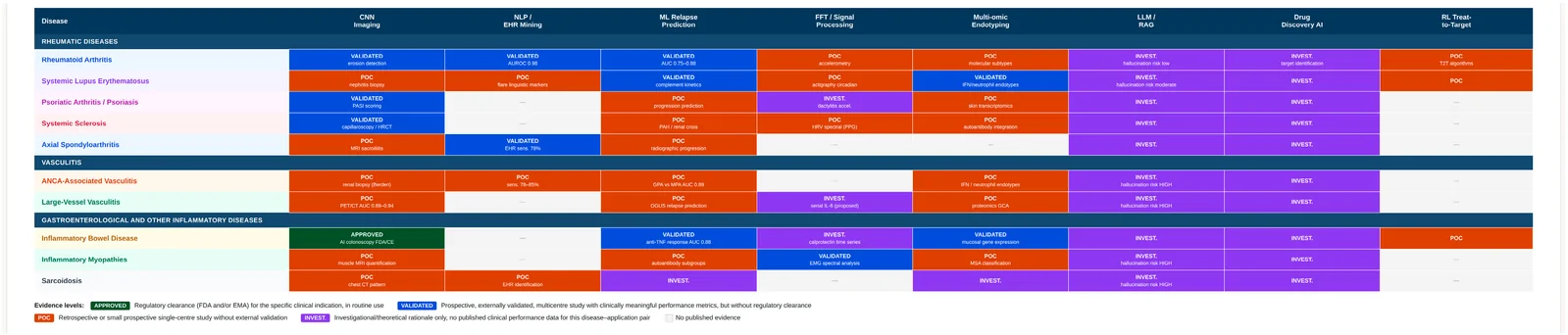
AI maturity heatmap across immune-mediated inflammatory diseases and clinical application domains. Each cell represents the current evidence level for the intersection of a specific disease entity and AI modality, assigned as described in the Literature Search and Selection Strategy (see main text); where evidence for a given disease–application pair was mixed, the more conservative category was assigned. LLM hallucination risk annotation reflects the estimated risk of factually incorrect AI-generated content, which is highest in rare inflammatory diseases with limited training data representation. APPROVED = regulatory clearance (FDA and/or EMA) for the specific clinical indication, in routine use; VALIDATED = prospective, externally validated, multicentre study with clinically meaningful performance metrics, but without regulatory clearance; POC = retrospective or small prospective single-centre study without external validation; INVEST. = investigational/theoretical rationale only, no published clinical performance data for this disease–application pair. CNN, convolutional neural network; NLP, natural language processing; EHR, electronic health record; ML, machine learning; FFT, Fast Fourier Transform; LLM, large language model; RAG, retrieval-augmented generation; RL, reinforcement learning; T2T, treat-to-target; PASI, Psoriasis Area and Severity Index; HRCT, high-resolution CT; PAH, pulmonary arterial hypertension; HRV, heart rate variability; PPG, photoplethysmography; EMG, electromyography; MSA, myositis-specific autoantibodies; IFN, interferon; GPA, granulomatosis with polyangiitis; MPA, microscopic polyangiitis; OGUS, OMERACT GCA Ultrasonography Score.

This is a narrative, clinically oriented review reflecting the authors’ expertise and clinical perspective, rather than a systematic review; no formal search protocol, dual independent screening, or PRISMA-style documentation of records identified/excluded was used, and this is an acknowledged limitation relative to a systematic review. Relevant literature was identified through non-systematic searches of PubMed/MEDLINE and Google Scholar combining disease-specific terms (e.g., rheumatoid arthritis, systemic lupus erythematosus, ANCA-associated vasculitis, inflammatory bowel disease, psoriatic arthritis, systemic sclerosis, inflammatory myopathies, sarcoidosis) with technology-specific terms (artificial intelligence, machine learning, deep learning, natural language processing, Fourier transform, wearable/accelerometry, large language model), supplemented by the authors’ clinical and methodological expertise and by review of the reference lists of identified papers. Study selection and the level of evidentiary weight given to each study were based on the authors’ judgement rather than on pre-specified inclusion/exclusion criteria; peer-reviewed original studies were generally prioritised over abstracts or preprints, and this hierarchy is reflected in the evidence-maturity categories introduced below (Figure 1 and Table S1) and applied throughout the manuscript.

This review is organised around three core themes: (i) AI/ML for diagnostic support and relapse/flare prediction across IMIDs (established and near-term applications, addressed in the sections “Machine Learning in Diagnostic Rheumatology and Inflammatory Disease” and “Artificial Intelligence for Relapse Prediction and Disease Monitoring”); (ii) Fourier transform-based signal processing as a novel, disease-agnostic monitoring modality (section “Fourier Transform Analysis and Biological Signal Processing”), presented in depth because it has not previously been synthesised for IMIDs; and (iii) large language models and the hallucination/safety problem in rare inflammatory disease (section “Large Language Models, NLP, and the Hallucination Problem”). Disease-specific findings are summarised concisely in Table S1 and Figure 1 and in the section “Artificial Intelligence in Specific Inflammatory Disease Entities”, which is intended as a reference map rather than an exhaustive account of each entity.

## 3. Machine Learning in Diagnostic Rheumatology and Inflammatory Disease

The diagnostic process in rheumatology and inflammatory disease medicine is inherently pattern-based—integrating clinical features, laboratory data, imaging findings, endoscopic results, and histopathological data into a probabilistic judgment. Many IMIDs share overlapping clinical and laboratory features, and their insidious onset means that differential diagnosis can be genuinely challenging, particularly in early or undifferentiated presentations. ML algorithms, trained on large datasets of labeled cases, can complement and in selected contexts enhance this pattern recognition capacity, offering particular value in settings where multiple data streams must be integrated simultaneously or where access to specialist expertise is limited.

### 3.1. Image Analysis Across Inflammatory Diseases

Convolutional neural networks (CNNs) have demonstrated remarkable performance in the analysis of imaging across multiple IMIDs and modalities. In plain radiography, ML models have achieved accuracy comparable to experienced rheumatologists in detecting erosions, joint space narrowing, and structural damage progression in rheumatoid arthritis [2]. In ultrasound, deep learning algorithms detect synovial hypertrophy, power Doppler signal, and erosions with sensitivities approaching those of expert sonographers [3]. In MRI, AI-assisted segmentation of synovial tissue, bone marrow edema, and cartilage has enabled quantitative assessment of inflammation surpassing conventional scoring systems such as RAMRIS [4]. In systemic sclerosis, AI analysis of HRCT has enabled automated quantification of interstitial lung disease extent and pattern recognition of fibrotic versus inflammatory components [5].

In inflammatory bowel disease, AI-assisted colonoscopy using real-time CNN analysis of endoscopic images has demonstrated accuracy for mucosal lesion detection approaching that of expert endoscopists in validation studies, with regulatory clearance obtained for a subset of systems, for endoscopic disease activity assessment, and histological remission prediction [6,7]. Several AI-assisted colonoscopy systems have received regulatory clearance in the United States and Europe, representing the most clinically advanced examples of AI implementation in inflammatory disease management [8]. In psoriasis, CNN-based analysis achieves dermatologist-level PASI scoring [9,10]. Nailfold capillaroscopy in systemic sclerosis has been transformed by ML-based automated pattern recognition, enabling standardized capillaroscopic pattern classification [11,12]. In vasculitis, AI-assisted analysis of PET/CT and vessel wall MRI has demonstrated proof-of-concept capacity to quantify vascular inflammation with precision exceeding human expert assessment [13].

### 3.2. Laboratory Data, Molecular Phenotyping, and Multi-Omic Integration

In SLE, unsupervised clustering algorithms have identified distinct molecular endotypes with different clinical trajectories and treatment responses [14,15]. In AAV, transcriptomic profiling combined with ML-based feature selection has identified gene expression signatures distinguishing PR3-ANCA from MPO-ANCA disease [16]. In IBD, ML analysis of mucosal gene expression profiles has identified molecular subtypes predicting response to anti-TNF therapy [17,18,19]. In systemic sclerosis, ML-based integration of autoantibody profiles, nailfold capillaroscopy patterns, and clinical variables has improved prediction of interstitial lung disease progression and scleroderma renal crisis risk [20]. In inflammatory myopathies, ML classification of myositis-specific autoantibody profiles has enabled automated assignment to clinical-serological subgroups with therapeutic and prognostic implications [21,22].

Natural language processing (NLP) applied to electronic health records has achieved AUROC values of up to 0.98 for RA identification [23], sensitivity of 95% and positive predictive value of 87% for outcome measure extraction from over 34 million clinical notes [24], and sensitivity of 78% with specificity of 94% for axial SpA identification [2]—representing validated clinical applications increasingly integrated into real-world EHR systems.

### 3.3. Endoscopy, Histopathology, and Procedural AI

In IBD, real-time AI-assisted colonoscopy systems have demonstrated superior polyp detection rates, more accurate endoscopic disease activity scoring, and improved prediction of histological remission. In rheumatology, ML-based analysis of synovial histopathology has identified patterns—including lymphoid aggregates, lining layer hyperplasia, and fibroblast activation—correlating with treatment response to biologics [2]. In inflammatory myopathies, automated muscle biopsy image analysis enables standardized quantification of inflammatory infiltrate density, fiber necrosis, and regeneration [25].

## 4. Artificial Intelligence for Relapse Prediction and Disease Monitoring

The prediction of disease relapse and optimization of treatment monitoring represent the most clinically impactful potential applications of AI across the IMID spectrum. ML models trained on longitudinal datasets integrating clinical, laboratory, imaging, endoscopic, and patient-reported data offer the potential to identify patterns of impending relapse with precision exceeding individual markers or composite scores.

### 4.1. Relapse Prediction in Rheumatoid Arthritis and Spondyloarthritis

In rheumatoid arthritis, supervised ML algorithms including random forests, gradient boosting machines, and support vector machines have been trained on longitudinal cohort data to predict flare risk at the individual patient level. Key predictive features include ACPA titer trajectories, CRP kinetics, power Doppler ultrasound scores, and patient-reported pain and fatigue scores. ML models consistently outperform single-marker approaches with AUC values ranging from 0.75 to 0.88 in validation cohorts [26,27]. Integration of wearable device data has further improved predictive performance [28,29]. In axial spondyloarthritis, ML models integrating MRI sacroiliitis scores, CRP trajectories, and HLA-B27 status have demonstrated superior prediction of radiographic progression [2,30].

### 4.2. Monitoring in Systemic Lupus Erythematosus

In SLE, ML approaches integrating serological markers (anti-dsDNA, complement C3/C4), haematological indices, urinary biomarkers, and patient-reported outcomes have identified temporal patterns preceding clinical flares by weeks [31,32]. Deep learning models have demonstrated that sequential complement normalization and re-elevation patterns are among the strongest predictors of impending flares, and temporal biomarker trajectories—including anti-dsDNA kinetics and complement dynamics—represent a promising early-warning signal that requires prospective validation before it can be considered clinically actionable [31].

### 4.3. Monitoring in Inflammatory Bowel Disease

In IBD, ML models integrating faecal calprotectin trajectories, endoscopic scores, serum drug levels, and anti-drug antibody profiles have demonstrated superior prediction of loss of response to biologic therapy, potentially enabling pre-emptive dose optimization [6,33]. Radiomics applied to CT and MRI enterography enables non-invasive transmural disease activity assessment with AUC values of 0.80–0.85 in validation cohorts [34,35].

### 4.4. Treat-to-Target Strategies and Reinforcement Learning

Reinforcement learning algorithms—in which models learn optimal decision strategies through iterative reward-based feedback—have been proposed as a promising framework for personalizing treatment escalation and de-escalation decisions across IMIDs, though clinical validation in rheumatology and gastroenterology remains limited to proof-of-concept studies. While clinical validation remains limited, reinforcement learning frameworks have been proposed for dynamic treatment optimization in IMIDs, with simulation studies suggesting potential to reduce cumulative glucocorticoid exposure and improve treat-to-target adherence compared to fixed protocol approaches.

## 5. Fourier Transform Analysis and Biological Signal Processing in Inflammatory Diseases

Fourier transform-based signal processing is not a novelty in medicine—it is, in fact, one of the most pervasive mathematical tools in modern clinical practice, operating largely invisibly at the foundation of technologies that clinicians use daily. Magnetic resonance image reconstruction from raw k-space data relies fundamentally on the Fast Fourier Transform for the mathematical reconstruction of spatial images from frequency-domain data [36]. In cardiology, spectral analysis of electrocardiographic signals and heart rate variability via FFT is a well-established method contributing to arrhythmia characterization, autonomic nervous system assessment, and risk stratification in heart failure [37]. In clinical neurophysiology, EEG spectral analysis—decomposing brain electrical activity into delta, theta, alpha, beta, and gamma frequency bands via FFT—is widely used in epilepsy monitoring, sleep staging, and encephalopathy assessment, typically alongside time-domain and other quantitative EEG methods. In sleep medicine, polysomnographic signal processing relies extensively on FFT-based frequency decomposition [38,39]. In audiology, brainstem auditory evoked potentials and otoacoustic emissions are commonly characterised through FFT-based spectral analysis [40].

The applications described below fall into two distinct evidentiary categories, which we distinguish explicitly throughout this section: (i) established, decades-old clinical uses of FFT in MRI, cardiology, EEG, sleep medicine, and audiology, cited above only to establish mathematical and technical precedent, and not themselves under evaluation in this review; and (ii) IMID-specific applications, most of which are proof-of-concept or purely proposed extensions and have not undergone the prospective, multicentre validation that underlies the established uses in (i). The mathematical maturity of FFT in established domains does not imply clinical effectiveness in IMIDs, which remains to be demonstrated.

**Figure 2:**
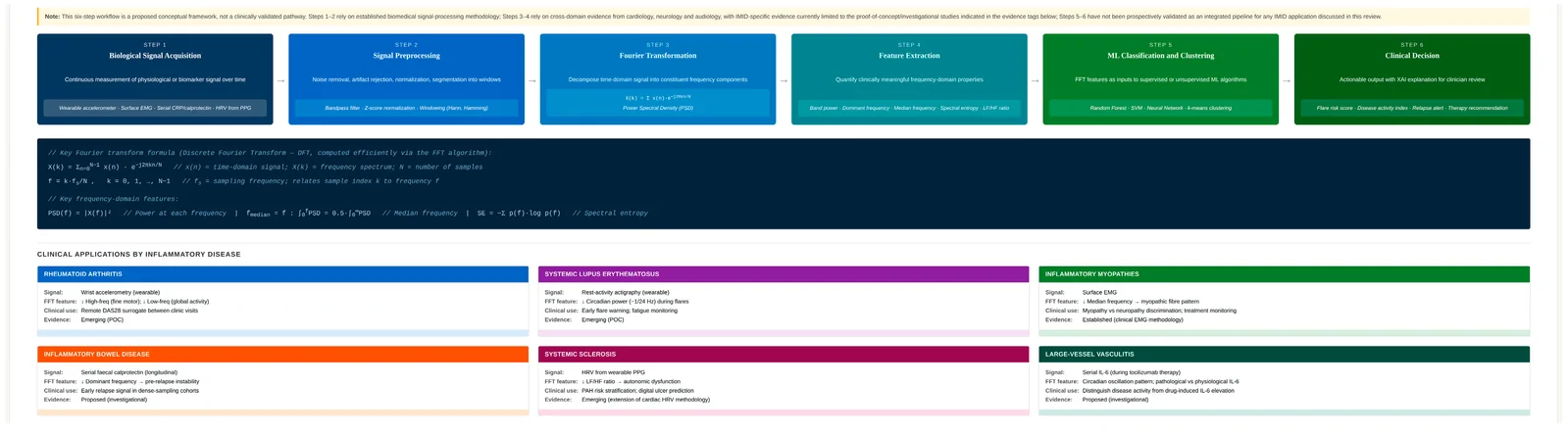
The FFT-ML pipeline for biological signal analysis in inflammatory diseases. Arrows indicate the sequential order of the six processing steps, from raw signal acquisition to clinician-facing clinical decision support. This six-step workflow is a proposed conceptual framework, not a clinically validated pathway: Steps 1–2 rely on established biomedical signal-processing methodology; Steps 3–4 rely on cross-domain evidence from cardiology, neurology and audiology, with IMID-specific evidence currently limited to the proof-of-concept/investigational studies indicated in the lower panels; Steps 5–6 have not been prospectively validated as an integrated pipeline for any IMID application discussed in this review. Lower panels illustrate disease-specific applications across rheumatoid arthritis, systemic lupus erythematosus, inflammatory myopathies, inflammatory bowel disease, systemic sclerosis, and large-vessel vasculitis, with evidence levels for each application. FFT, Fast Fourier Transform; ML, machine learning; XAI, explainable artificial intelligence; EMG, electromyography; HRV, heart rate variability; PPG, photoplethysmography; PSD, power spectral density; LF/HF, low-frequency/high-frequency ratio; PAH, pulmonary arterial hypertension; DAS28, Disease Activity Score 28; POC, proof-of-concept.

The application of these same mathematical principles to the biological signals generated by patients with immune-mediated inflammatory diseases—accelerometry, electromyography, heart rate variability, and longitudinal biomarker time series—represents a natural and logical extension of established methodology into a domain where it has been underexplored. The novelty lies not in the mathematics, which is decades old and clinically validated across multiple specialties, but in the clinical questions being asked and the biological signals being analyzed. The proposed end-to-end analysis pipeline, from signal acquisition to clinical decision support, is summarised in Figure 2.

### 5.1. Fundamentals of Fourier Transform Analysis for the Clinician

The Fourier transform (FT) represents a time-varying signal in terms of its frequency content by expressing it as a superposition of sinusoidal components with different frequencies, amplitudes, and phases. In its discrete form—the Discrete Fourier Transform (DFT), computed efficiently via the Fast Fourier Transform (FFT) algorithm—it enables the analysis of digitized biological signals, transforming data from the time domain into the frequency domain. This frequency-domain representation can reveal periodic structures in the data that may not be apparent from direct inspection of the original time series—for example, identifying whether a biomarker fluctuates with a circadian rhythm, a weekly pattern, or a longer inflammatory cycle [41,42].

In the context of inflammatory diseases, FFT-based analysis has three principal candidate clinical applications: decomposition of physiological signals into frequency components reflecting pathological neuromuscular or vascular states; identification of oscillatory patterns in longitudinal biomarker time series potentially corresponding to subclinical disease cycles; and pre-processing of biological signals prior to ML classification, where frequency-domain features have been reported to improve classifier performance relative to raw time-domain data in several non-IMID applications (see “Integration of FFT Features with Machine Learning Pipelines” below).

### 5.2. Accelerometry and Wearable Devices Across Inflammatory Diseases

Wearable accelerometers generate continuous time-series data on movement, physical activity, and rest-activity cycles. In rheumatoid arthritis, FFT analysis of accelerometry data has identified characteristic frequency signatures of disease-associated movement impairment, including reduced high-frequency components corresponding to fine motor activities and altered low-frequency patterns reflecting overall physical activity reduction—features demonstrating superior correlation with DAS28 and HAQ compared to simple step-count metrics [28,29]. In SLE, wearable-derived rest-activity rhythms correlate with fatigue severity and flare risk—circadian rhythm disruption has been identified as a predictor of disease progression with an AUC of 0.76 [43], and FFT-based quantification of spectral power in the circadian frequency band represents a promising investigational approach for continuous remote monitoring. In IBD, accelerometry-derived physical activity patterns correlate with disease activity scores and patient-reported fatigue [44], and FFT-based frequency decomposition of these signals represents a promising investigational approach for remote disease monitoring. In psoriatic arthritis, FFT analysis of hand movement accelerometry shows promise for remote assessment of dactylitis and finger joint inflammation [45]. In systemic sclerosis, FFT analysis of heart rate variability signals derived from wearable photoplethysmographs has identified autonomic dysfunction patterns correlating with pulmonary arterial hypertension risk and digital ulcer burden—an extension of the well-established use of HRV spectral analysis in cardiovascular medicine to a rheumatological context [46].

### 5.3. Electromyographic Signal Analysis in Inflammatory Myopathies and Vasculitic Neuropathy

In inflammatory myopathies, FFT-based spectral analysis of EMG signals has enabled quantitative characterization of myopathic versus neuropathic changes, detecting shifts in the median frequency of the power spectrum reflecting alterations in muscle fiber conduction velocity and motor unit composition—an application of EMG spectral analysis techniques established in neuromuscular medicine [47]. These spectral features demonstrate diagnostic utility in distinguishing inflammatory from non-inflammatory myopathies and in monitoring treatment response [21,48]. In vasculitic neuropathy, spectral analysis of nerve conduction signals—an extension of established quantitative neurophysiology techniques—may enable more precise quantification of axonal loss and demyelination patterns than conventional amplitude and velocity measurements alone, though prospective validation in vasculitic neuropathy cohorts is required [47].

### 5.4. Oscillatory Patterns in Biomarker Time Series

Inflammatory markers including CRP, ESR, faecal calprotectin, and cytokine levels exhibit complex temporal dynamics including circadian rhythms, ultradian oscillations, and longer-period cycles corresponding to subclinical disease fluctuations [49,50]. IL-6 exhibits well-documented circadian oscillatory behavior driven by the HPA axis and the autonomic nervous system—a phenomenon amenable to FFT-based quantification [51,52]. In IBD, we hypothesise—as a conceptual extension of FFT-based biomarker dynamics analysis from endocrinology, and without direct supporting evidence in IBD at present—that oscillatory behaviour of faecal calprotectin over time, with shifts toward lower-frequency dominant oscillations, could represent a marker of mucosal disease stability; this remains an untested hypothesis requiring prospective evaluation. We propose that analogous approaches applied to serial IL-6 and CRP measurements in RA and LVV may reveal subclinical inflammatory cycles that are invisible to episodic clinic-based assessment; prospective validation of these approaches in rheumatological cohorts represents an important investigational priority.

### 5.5. Integration of FFT Features with Machine Learning Pipelines

FFT-derived frequency-domain features—spectral power in defined frequency bands, dominant frequency, spectral entropy—provide compact, informative representations that often improve ML classifier performance when used as inputs instead of raw time-series data [53]. This approach is well-established in cardiac arrhythmia detection [54], epilepsy prediction [38], and sleep staging [55], and is increasingly being translated to IMID applications including automated flare detection [28,29] from wearable accelerometry data, classification of inflammatory versus mechanical pain patterns from movement signals, and identification of autonomic dysfunction in systemic sclerosis from heart rate variability analysis [20].

## 6. Artificial Intelligence in Specific Inflammatory Disease Entities

### 6.1. Vasculitis

Vasculitis represents a particularly challenging domain given the heterogeneity of presentations across vessel calibers and the rarity of individual syndromes. In AAV, ML algorithms applied to clinical, serological, and histopathological data have shown promise for improving diagnostic classification—including the discrimination of GPA from MPA beyond individual ANCA specificity—with proof-of-concept evidence accumulating across registry datasets [13]. Deep learning analysis of renal biopsy histopathology has enabled automated Berden schema classification approaching expert nephropathologists, with a prediction accuracy of 93% in a multicentre European cohort [56]. NLP algorithms applied to EHR clinical notes have achieved sensitivity of 78–85% for identifying undiagnosed AAV [57]. In large-vessel vasculitis, deep learning applied to 18F-FDG PET/CT has achieved AUC values of 0.89–0.94 for distinguishing active from inactive disease. A 2025 systematic review identified 47 AI applications in vasculitis across diagnostic, prognostic, and therapeutic domains [13].

### 6.2. Systemic Lupus Erythematosus

ML has been applied to lupus nephritis classification from renal biopsy images, achieving automated ISN/RPS class assignment approaching expert nephropathologists [58]. In neuropsychiatric SLE, ML analysis of brain MRI features and cerebrospinal fluid biomarkers has demonstrated capacity to distinguish inflammatory from non-inflammatory neurological involvement [59,60]. Multi-omic ML approaches have identified molecular SLE endotypes—including type I interferon-high, complement-low, and neutrophil-dominant subtypes—with distinct therapeutic vulnerabilities that may guide biological therapy selection [14,15].

### 6.3. Inflammatory Bowel Disease

IBD represents the most clinically advanced domain for AI implementation among IMIDs, with regulatory-approved AI-assisted colonoscopy systems already in clinical use—while most other AI applications discussed in this review remain at the proof-of-concept or investigational stage (Table S1, Figure 1). ML models integrating pre-treatment mucosal gene expression profiles, microbiome composition, serum proteomics, and clinical variables have demonstrated AUC values of 0.80–0.88 for predicting anti-TNF response. Deep learning analysis of CT and MRI enterography enables non-invasive transmural disease activity assessment correlating with endoscopic and histological findings [17,18,19].

### 6.4. Psoriatic Arthritis and Psoriasis

CNN-based analysis of dermoscopic images achieves dermatologist-level PASI scoring and treatment response assessment [9,10]. In psoriatic arthritis, ML models integrating skin disease severity, dactylitis scores, enthesitis measures, and imaging findings have demonstrated superior prediction of radiographic progression [61,62]. ML analysis of clinical and molecular features from psoriasis patients has identified subtypes with differential joint involvement risk, potentially enabling identification of patients who would benefit from early rheumatological screening [63].

### 6.5. Systemic Sclerosis

ML-based automated nailfold capillaroscopy analysis has moved beyond simple pattern recognition to deep learning models classifying capillaroscopic images according to the standardised “early/active/late” scleroderma pattern, achieving agreement with expert raters comparable to inter-observer agreement between experts themselves, and automated quantification of capillary density and morphological abnormalities (giant capillaries, microhaemorrhages, avascular areas) that reduces operator dependency and inter-centre variability—a longstanding limitation of manual capillaroscopy scoring [11,12]. Integration of automated capillaroscopy output with autoantibody profiles and clinical variables has been proposed as a composite risk score for progression from isolated Raynaud’s phenomenon/very early SSc to definite disease (see VEDOSS discussion below). AI analysis of HRCT patterns distinguishes usual interstitial pneumonia from non-specific interstitial pneumonia and quantifies fibrotic burden [5,20]. ML models integrating autoantibody profiles, nailfold capillaroscopy patterns, and pulmonary function trajectories have improved prediction of scleroderma renal crisis and pulmonary arterial hypertension [64,65].

A clinically important but AI-unaddressed application area is risk stratification in patients meeting VEDOSS (Very Early Diagnosis of Systemic Sclerosis) criteria—Raynaud’s phenomenon with SSc-specific autoantibodies, puffy fingers, and/or a scleroderma pattern on nailfold capillaroscopy, but without definite fibrotic disease. Conventional statistical analyses of large VEDOSS cohorts have identified combinations of these features as the strongest predictors of progression to definite SSc [66], yet to our knowledge no published study has applied machine learning specifically within a VEDOSS population. Given that ML-based models integrating clinical and serological variables have already demonstrated good discriminative performance for outcome prediction in established SSc [67], extending such approaches—combined with the automated capillaroscopy classification described above—to the VEDOSS population to predict conversion to definite SSc represents a concrete and currently unmet research opportunity, rather than an existing validated application.

### 6.6. Inflammatory Myopathies and Sarcoidosis

In inflammatory myopathies, ML classification of myositis-specific autoantibody profiles combined with clinical and histopathological data has improved subgroup assignment accuracy, with direct therapeutic implications—anti-MDA5 positive dermatomyositis requires urgent ILD assessment and aggressive immunosuppression, while anti-TIF1γ positive disease warrants systematic malignancy screening [21,22]. In sarcoidosis, ML analysis of chest CT features has improved characterization and diagnosis of pulmonary involvement, with deep learning approaches enabling automated distinction from other interstitial lung diseases [68].

## 7. Large Language Models, NLP, and the Hallucination Problem in Inflammatory Diseases

Large language models (LLMs)—transformer-based neural networks trained on vast corpora of text data—demonstrate remarkable generalization across diverse clinical scenarios including differential diagnosis generation, clinical note summarization, guideline adherence assessment, and patient communication [69,70]. Early evaluations on rheumatological board examination questions have demonstrated performance at or above the level of certified rheumatologists on standardized assessments [71]. NLP techniques applied to EHR have achieved AUROC values of up to 0.98 for RA identification, sensitivity of 95% for outcome measure extraction from over 34 million clinical notes, and sensitivity of 78% with specificity of 94% for axial SpA identification [2,23,24].

### The Hallucination Problem: Critical Implications for Rare Inflammatory Diseases

A critical limitation of LLMs in clinical inflammatory disease management is the phenomenon of hallucination: the tendency of LLMs to generate clinically plausible but factually incorrect information, presented with apparent confidence. In rheumatology, hallucination risk varies significantly according to disease prevalence and available training data: it is relatively contained in high-prevalence conditions such as rheumatoid arthritis, but becomes clinically critical in rare diseases—including SAPHO syndrome, adult-onset Still’s disease, and systemic vasculitides—where training data are scarce and model outputs are correspondingly less reliable. Hallucination in rare disease contexts carries direct patient safety implications, as erroneous treatment recommendations presented with apparent confidence may lead to inappropriate immunosuppression or dangerous drug combinations.

Retrieval-Augmented Generation (RAG)—anchoring LLM outputs to verified external sources such as EULAR and ACR guidelines—has emerged as the most promising mitigation strategy, significantly reducing hallucination rate. A recent study integrating 74 EULAR and ACR guidelines into a RAG framework demonstrated system preference by rheumatologist evaluators in 71–74% of comparisons versus a baseline LLM [72]. The clinical deployment of LLMs in inflammatory disease management should be considered investigational pending prospective validation, and RAG integration should be considered a minimum safety requirement for any clinical implementation.

RAG substantially reduces but does not eliminate hallucination risk: retrieval failures, citation of superseded guideline versions, misalignment between retrieved context and the specific clinical question, and inappropriate extrapolation from retrieved evidence to an individual patient remain possible. Safe clinical deployment therefore requires, at minimum: mandatory clinician review of all AI-generated recommendations before action is taken; a clear accountability framework specifying that the treating clinician, not the model, retains clinical and legal responsibility; a defined mechanism for continuous monitoring of model outputs against clinical outcomes, with periodic re-validation; a process for timely incorporation of updated EULAR/ACR/ECCO guidelines into the retrieval corpus, with version control; and output traceability, whereby every AI-generated recommendation is linked to its specific source passages so that clinicians can verify the underlying evidence before relying on it.

Beyond clinical imaging, omics, and EHR data, AI/ML methods are increasingly applied to assess the quality and reliability of patient-facing health information circulating outside clinical settings, including social media content; interpretable ML-based quality assessment of weight-management short-form video content on TikTok and Bilibili illustrates this emerging application of NLP and content-analysis models to real-world public health communication [73].

## 8. Limitations, Bias, and Ethical Considerations

### 8.1. Data Quality, Representativeness, and Algorithmic Bias

The performance of any ML algorithm is fundamentally constrained by the quality, quantity, and representativeness of the training data. In inflammatory disease management, training datasets are frequently derived from tertiary referral centers in high-income countries, introducing systematic selection biases. Racial, ethnic, and socioeconomic underrepresentation in IMID registries is directly inherited by ML models, potentially generating algorithms that perform well in populations they were trained on but fail in others—and risk automating existing diagnostic disparities [74,75].

### 8.2. The Interpretability Challenge: Black Box Medicine and the Epistemology of Observation

The most powerful ML architectures achieve their performance at the cost of interpretability. Deep learning models operate as “black boxes” whose internal decision logic is opaque—a feature particularly problematic in inflammatory disease management where treatment decisions carry significant toxicity risks and where the clinical rationale for algorithmic recommendations must be communicable to patients. Explainable AI (XAI) methods—including SHAP values, LIME, and attention visualization—provide post-hoc explanations of individual model predictions and should be considered a minimum requirement for clinical AI deployment [76]. Until recently, such methodological capabilities were not even conceivable in clinical medicine, and their emergence should be approached with both enthusiasm and intellectual humility.

Beyond the interpretability challenge, AI and signal processing methods raise a more fundamental epistemological question: not merely how we interpret clinical phenomena, but what we are capable of observing in the first place. The oscillatory patterns, frequency-domain signatures, and subclinical inflammatory cycles described in this review were not previously misinterpreted—they were simply invisible to the observational tools available to clinicians and researchers. Just as the microscope did not merely reinterpret inflammation but revealed an entirely new level of biological organization, AI-driven signal analysis may reveal layers of disease activity that alter not only our interpretation but the foundational observations upon which our understanding is built. This epistemological humility should accompany enthusiasm for AI as a clinical tool.

### 8.3. Regulatory Framework and Clinical Validation

The European AI Act (2024) classifies AI systems used for medical diagnosis as high-risk applications subject to mandatory conformity assessment and post-market surveillance [77,78]. Despite these regulatory developments, the vast majority of published AI studies in inflammatory diseases are retrospective, single-center, and evaluated on internal test sets—a validation approach that systematically overestimates real-world performance [79]. The regulatory-approved AI colonoscopy systems in IBD represent the current gold standard for clinical AI validation and should serve as a model for other IMID applications [6,8].

### 8.4. Privacy, Data Governance, and the Human-AI Interface

Federated learning—enabling collaborative model training across institutions without centralizing patient data—represents a promising solution particularly relevant for rare IMIDs where no single institution can accumulate sufficient training data [80]. The WHO’s guidance on ethics and governance of AI for health (2021) identifies six core principles—transparency, accountability, inclusiveness, empowerment, sustainability, and protection of privacy—that should guide AI development in inflammatory disease medicine [81]. A fundamental principle for AI integration in clinical practice is that of augmentation rather than replacement: AI tools are most appropriately conceptualized as decision support systems that enhance clinical judgment while leaving the final decision in the hands of the responsible clinician.

## 9. Future Directions: Toward Precision Inflammatory Disease Medicine

### 9.1. Digital Biomarkers and Continuous Remote Monitoring

The proliferation of wearable sensors is generating unprecedented volumes of continuous physiological data from patients with IMIDs. FFT-based signal processing will play a central role in extracting clinically meaningful features from these high-frequency data streams. The identification of frequency-domain signatures of disease activity—detectable weeks before clinical flares—could enable pre-emptive therapeutic adjustments that fundamentally change the therapeutic paradigm from reactive to predictive medicine across the IMID spectrum.

### 9.2. Federated Learning and International Data Collaboration

The rarity of many IMIDs means that no single institution can accumulate training datasets of sufficient size for robust, generalizable AI models. International initiatives such as the EUVAS network, LUMINA lupus cohort, CORRONA registry, and IBD Biobank represent existing infrastructures adaptable for federated AI development, enabling model training on tens of thousands of patients while preserving institutional data sovereignty.

### 9.3. AI-Assisted Drug Discovery and Repurposing

Graph neural networks applied to protein interaction networks have identified candidate drug targets in inflammatory pathways relevant to multiple IMIDs. Generative AI models are being used to generate novel small molecule candidates with predicted anti-inflammatory activity. AI-driven drug repurposing has generated testable hypotheses for refractory SSc-ILD, vasculitis, and rare autoimmune conditions, with some predictions already under clinical investigation.

### 9.4. AI, the “Atypical Patient”, and the Limits of Inherited Clinical Knowledge

**Figure 3:**
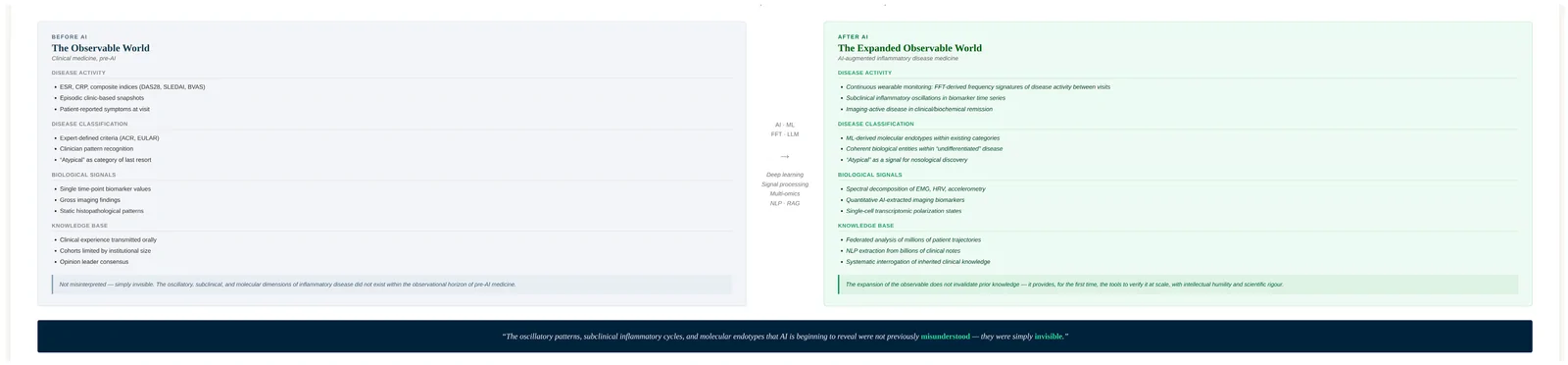
The Observability Gap in inflammatory disease medicine. The left panel illustrates the observable domain of pre-AI clinical medicine — episodic, snapshot-based, and constrained by the resolution of available instruments. The right panel depicts the expanded observable world enabled by artificial intelligence, machine learning, Fourier transform-based signal processing, and large language models. The central arrow denotes the transformation from pre-AI to AI-augmented observational capacity, enabled by the listed methods (deep learning, signal processing, multi-omics, NLP, RAG). The transition is not a replacement of prior knowledge but an epistemological expansion: phenomena that were previously invisible become observable, enabling both nosological discovery and the systematic verification of clinical knowledge transmitted through generations of medical tradition. AI, artificial intelligence; ML, machine learning; FFT, Fast Fourier Transform; LLM, large language model; NLP, natural language processing; RAG, retrieval-augmented generation; HRV, heart rate variability; EMG, electromyography.

Every experienced clinician carries a cohort of patients informally described as “atypical” or “difficult to classify”—patients who do not fit established diagnostic categories despite extensive investigation. A transformative implication of AI-driven observational tools is that these patients may not be genuinely atypical, but rather reflect the boundaries of current nosological knowledge: the “atypical patient” may be an artefact of incomplete disease taxonomy rather than a true biological outlier. ML-based unsupervised clustering applied to large, phenotypically diverse IMID cohorts may reveal that what clinicians currently label as undifferentiated, seronegative, or atypical disease in fact represents coherent biological entities—with distinct molecular signatures, imaging phenotypes, and treatment responses—that pre-AI classification systems are simply unable to resolve. The systematic collection and ML-based phenotyping of these currently underrepresented “atypical” cases represents a high-yield research priority (Figure 3).

This challenge extends beyond individual patients to the knowledge base of clinical medicine itself. Much of what clinicians “know” about the natural history and prognosis of inflammatory diseases has been transmitted through clinical teaching, departmental tradition, and opinion-leader consensus, rather than derived from systematic analysis of large, prospectively collected populations—a mode of transmission that, while valuable, is subject to selection bias and individual cognitive heuristics. AI now offers, for the first time, the technical capacity to interrogate this inherited knowledge systematically and at scale. The appropriate response is neither uncritical enthusiasm nor defensive rejection, but intellectual humility: a willingness to hold established knowledge open to revision while first understanding it before questioning it. Large-scale, AI-driven observational studies designed to interrogate—not merely confirm—existing knowledge represent an important emerging research paradigm; some received wisdom will be confirmed, some revealed as incomplete, and some overturned.

### 9.5. Toward a Precision Inflammatory Disease Framework

The ultimate vision is a precision inflammatory disease framework in which each patient’s diagnosis, treatment selection, monitoring strategy, and tapering schedule are informed by an integrated analysis of their individual molecular profile, imaging phenotype, digital biomarker trajectory, and clinical history—analogous to precision oncology. Realizing this vision will require coordinated investment in interoperable clinical data infrastructures, prospective AI validation studies, regulatory frameworks, and cultivation of AI literacy among practising clinicians and trainees.

## 10. Conclusions

Artificial intelligence and machine learning are beginning to transition from experimental tools toward clinically relevant applications in selected domains, while the majority of applications discussed in this review remain investigational. The convergence of deep learning-based image analysis—including regulatory-approved AI endoscopy in IBD and proof-of-concept imaging AI in vasculitis and systemic sclerosis—with NLP-driven EHR mining, multi-omic biomarker discovery, and Fourier transform-based biological signal processing is creating a new paradigm of data-driven inflammatory disease management.

The Fourier transform approaches described in this review extend a mathematical framework already foundational in MRI physics, cardiac electrophysiology, and clinical neurophysiology into a new clinical domain. The novelty lies not in the mathematics but in the questions being asked: whether the oscillatory dynamics of inflammatory biomarkers, the frequency signatures of wearable-derived movement data, and the spectral characteristics of electromyographic signals contain clinically actionable information about disease activity that is invisible to conventional time-domain assessment. We propose that they do, and that prospective validation of FFT-based biomarker analysis in IMID cohorts represents an important and underexplored research priority.

The hallucination problem of large language models represents a critical safety challenge that is particularly acute in rare inflammatory diseases where training data are scarce. Retrieval-augmented generation offers a promising architectural solution but requires prospective clinical validation before widespread implementation [72].

Perhaps most significantly, this review argues that AI-driven methods do not merely improve the interpretation of existing clinical observations, but fundamentally expand the boundaries of what is observable. The oscillatory patterns, subclinical inflammatory cycles, and molecular endotypes that AI is beginning to reveal were not previously misunderstood—they were simply invisible. This epistemological shift invites a humble and courageous revisitation of clinical knowledge transmitted through generations of medical tradition: not to discard it, but to verify it with the rigour that immense computational capacity now makes possible. The patient who has always been described as “atypical” may, in the light of AI-driven nosological discovery, turn out to be the most informative of all.

The future of inflammatory disease medicine lies not in the replacement of clinical judgment by algorithmic decision-making, but in the augmentation of human expertise by AI tools that surface patterns, predictions, and insights that exceed unaided cognitive capacity. The clinician of the future will be a precision medicine practitioner—fluent in the language of molecular phenotypes, digital biomarkers, and AI-assisted diagnostics—while remaining, above all, a physician whose primary commitment is to the individual patient’s wellbeing and autonomy.

## Data Availability

No new data were created or analyzed in this study. Data sharing is not applicable to this article.

## Supplementary Materials

The following supporting information can be downloaded at: https://www.mdpi.com/article/10.3390/jcm15176864/s1, Table S1: Artificial Intelligence Applications in Immune-Mediated Inflammatory Diseases: Overview by Disease Entity and AI Modality; Table S2: Fourier Transform Analysis in Medicine: From Established Applications to Emerging and Proposed Uses in Immune-Mediated Inflammatory Diseases; Table S3: Machine Learning Algorithms in Immune-Mediated Inflammatory Diseases: Clinical Task, Disease Context, and Reported Performance; Table S4: Practical Guide for the Clinician: How to Critically Evaluate an AI Tool in Inflammatory Disease Medicine.
