# Supplementary material for: Artificial Intelligence and Machine Learning in Rheumatology and Systemic Inflammatory Diseases: From Pattern Recognition to Signal Analysis and Clinical Decision Support
Source: J Clin Med. 2026 Sep 4;15(17):6864. doi: 10.3390/jcm15176864 (PMC13566619; doi:10.3390/jcm15176864)
Supplement: Supplementary file 1 [file jcm-15-06864-s001.zip › Supplementary_Table_S1.pdf]

Supplementary Table S1. Artificial Intelligence Applications in Immune-Mediated Inflammatory Diseases: Overview by Disease Entity and AI Modality.

Disease / Application	CNN Imaging AI	NLP / EHR Mining	ML Predictive Models	FFT / Signal Processing	LLM / RAG	Multi-omic Endotyping
**RHEUMATOID ARTHRITIS**						
Erosion/damage detection (X-ray, US)	VALIDATED	—	—	—	—	—
Flare prediction	—	VALIDATED	VALIDATED	POC	INVEST.	POC
Patient identification (EHR)	—	VALIDATED	—	—	POC	—
Wearable monitoring (FFT)	—	—	POC	POC	—	—
**SYSTEMIC LUPUS ERYTHEMATOSUS**						
Nephritis biopsy classification	POC	—	—	—	—	—
Flare prediction	—	POC	VALIDATED	POC	INVEST.	POC
Molecular endotyping	—	—	—	—	—	VALIDATED
**VASCULITIS (AAV / LVV)**						
PET/CT activity quantification	POC	—	—	—	—	—
Halo sign detection (ultrasound)	POC	—	—	—	—	—
Patient identification (EHR)	—	POC	POC	—	INVEST.	POC
Renal biopsy (Berden classification)	POC	—	—	—	—	—
**INFLAMMATORY BOWEL DISEASE**						
AI-assisted colonoscopy	APPROVED	—	—	—	—	—
Anti-TNF response prediction	—	—	VALIDATED	INVEST.	—	VALIDATED
MRI/CT enterography radiomics	POC	—	VALIDATED	INVEST.	—	—
**PSORIATIC ARTHRITIS / PSORIASIS**						
PASI scoring (dermoscopy)	VALIDATED	—	—	—	—	—
Joint progression prediction	—	—	POC	INVEST.	—	POC
**SYSTEMIC SCLEROSIS**						
Nailfold capillaroscopy	VALIDATED	—	—	—	—	—
HRCT-ILD quantification	VALIDATED	—	POC	—	—	—
HRV / autonomic dysfunction (FFT)	—	—	POC	POC	—	—
PAH / renal crisis prediction	—	—	POC	—	—	POC
**INFLAMMATORY MYOPATHIES**						
Autoantibody subgroup classification	—	—	POC	—	INVEST.	POC
EMG spectral analysis (FFT)	—	—	POC	VALIDATED	—	—
Muscle MRI quantification	POC	—	—	—	—	—
**AXIAL SPONDYLOARTHRITIS**						
MRI sacroiliitis detection	POC	—	POC	—	—	—
Patient identification (EHR)	—	VALIDATED	—	—	POC	—

Evidence levels: **APPROVED** Regulatory clearance (FDA and/or EMA) for the specific clinical indication, in routine use **VALIDATED** Prospective, externally validated, multicentre study with clinically meaningful performance metrics, but without regulatory clearance **POC** Retrospective or small prospective single-centre study without external validation **INVEST.** Investigational/theoretical rationale only, no published clinical performance data for this disease–application pair — No published evidence

Category assignment reflects the highest level of evidence identified for each cell as of the literature search described in the manuscript; where evidence was mixed, the more conservative category was assigned.

Supplementary Table S1. Overview of artificial intelligence applications across major immune-mediated inflammatory diseases. AAV, ANCA-associated vasculitis; LVV, large-vessel vasculitis; IBD, inflammatory bowel disease; CNN, convolutional neural network; NLP, natural language processing; EHR, electronic health record; ML, machine learning; FFT, Fast Fourier Transform; LLM, large language model; RAG, retrieval-augmented generation; PASI, Psoriasis Area and Severity Index; HRCT-ILD, high-resolution CT-interstitial lung disease; HRV, heart rate variability; PAH, pulmonary arterial hypertension; EMG, electromyography; US, ultrasound; PET/CT, positron emission tomography/computed tomography.
