# Supplementary material for: Artificial Intelligence and Machine Learning in Rheumatology and Systemic Inflammatory Diseases: From Pattern Recognition to Signal Analysis and Clinical Decision Support
Source: J Clin Med. 2026 Sep 4;15(17):6864. doi: 10.3390/jcm15176864 (PMC13566619; doi:10.3390/jcm15176864)
Supplement: Supplementary file 1 [file jcm-15-06864-s001.zip › Supplementary_Table_S2.pdf]

Supplementary Table S2. Fourier Transform Analysis in Medicine: From Established Applications to Emerging and Proposed Uses in Immune-Mediated Inflammatory Diseases.

Medical Domain	Biological Signal	FFT Application	IMID Relevance	Evidence Level	Notes / References
**PART A — ESTABLISHED FFT APPLICATIONS IN CLINICAL MEDICINE**					
MRI Physics	K-space raw data	Reconstruction of spatial images from frequency-domain data	Foundation of all MRI-based IMID imaging (synovitis, ILD, sacroiliitis, VW-MRI)	ESTABLISHED	Mathematical basis of all clinical MRI; without FFT, MRI does not exist
Cardiology	ECG signal, Heart Rate Variability (HRV)	Spectral decomposition: LF/HF ratio, autonomic balance, arrhythmia detection	HRV spectral analysis extended to SSc autonomic dysfunction; FFT-based rhythm analysis in SLE pericarditis	ESTABLISHED	Standard clinical practice; HRV spectral indices validated for cardiovascular risk
Clinical Neurophysiology	EEG signal	Frequency band decomposition (delta, theta, alpha, beta, gamma)	Encephalopathy in NPSLE; CNS vasculitis monitoring; neurological complications of IMIDs	ESTABLISHED	Standard in epilepsy monitoring, sleep staging, encephalopathy assessment
Sleep Medicine	Polysomnography (EEG, EMG, ECG, respiration)	Multi-signal spectral decomposition for sleep staging	Sleep disturbance monitoring in RA, SLE, fibromyalgia comorbidity	ESTABLISHED	Regulatory-grade sleep staging algorithms rely on FFT-based features
Audiology	Otoacoustic emissions, ABR	Frequency-specific hearing threshold determination	Drug ototoxicity monitoring (MTX, aminoglycosides in septic vasculitis)	ESTABLISHED	Standard audiological diagnostic tool
Respiratory Medicine	Respiratory flow signals	Spectral analysis of breathing patterns, forced oscillation technique	Pulmonary monitoring in SSc-ILD, GPA respiratory involvement, asthma in EGPA	ESTABLISHED	FOT (ImpediMetry) uses FFT for respiratory resistance/reactance quantification
EMG (Neuromuscular)	Motor unit action potentials	Median frequency shift as marker of muscle fatigue and fiber composition	Inflammatory myopathy diagnosis and treatment monitoring; vasculitic neuropathy	ESTABLISHED	EMG spectral analysis standard in clinical neurophysiology laboratories
**PART B — EMERGING FFT APPLICATIONS IN INFLAMMATORY DISEASES**					
Rheumatology (RA)	Wrist/hand accelerometry (wearable)	FFT of movement time series: high-frequency fine motor components, low-frequency global activity	Disease activity monitoring between clinic visits; DAS28/HAQ correlation superior to step count	EMERGING	Proof-of-concept studies; prospective validation ongoing
Rheumatology (SLE)	Rest-activity rhythm (actigraphy)	Circadian power at ~1/24 Hz band; rhythm fragmentation index	Fatigue severity and flare risk correlation; circadian spectral power reduced during flares	EMERGING	Cross-sectional studies in SLE cohorts; longitudinal data limited
Gastroenterology (IBD)	Physical activity (wearable accelerometry)	FFT-derived activity frequency signatures correlating with disease burden	Fatigue monitoring in Crohn's and UC; remote activity assessment between colonoscopies	EMERGING	Early observational data; formal FFT-based IBD monitoring studies needed
Rheumatology (SSc)	Heart rate variability (wearable PPG)	LF/HF ratio, total spectral power, autonomic spectral indices	PAH risk stratification; digital ulcer burden correlation; autonomic neuropathy detection	EMERGING	Extension of established cardiac HRV methodology to SSc-specific outcomes
Neuromuscular / Myositis	Surface EMG	Median frequency shift; spectral entropy; myopathic vs neuropathic pattern discrimination	Inflammatory myopathy subtype differentiation; treatment response monitoring	EMERGING	Extension of established EMG spectral methodology to inflammatory myopathies
Rheumatology (PsA)	Finger/hand movement (wearable)	FFT of dactylitis-associated movement signatures	Remote assessment of dactylitis and finger joint inflammation between visits	EMERGING	Concept extrapolated from RA accelerometry literature; PsA-specific data limited
**PART C — PROPOSED/INVESTIGATIONAL FFT APPLICATIONS IN INFLAMMATORY DISEASES**					
Rheumatology / Gastroenterology	Serial biomarker measurements (CRP, ESR, calprotectin)	Oscillatory pattern detection: dominant frequency, spectral entropy of time series	Pre-relapse signal in RA, LVV, IBD; subclinical inflammatory cycle detection	PROPOSED	Theoretical framework; requires prospective dense-sampling longitudinal cohorts
Rheumatology (LVV)	Serial IL-6 measurements during tocilizumab therapy	Circadian oscillation characterization to distinguish physiological from pathological IL-6	Monitoring in GCA/TAK on tocilizumab where standard IL-6 interpretation is unreliable	PROPOSED	Based on established IL-6 circadian biology; FFT application to LVV-specific context proposed

Medical Domain	Biological Signal	FFT Application	IMID Relevance	Evidence Level	Notes / References
Multimodal	Combined wearable + biomarker streams	Multivariate FFT feature extraction as ML classifier inputs	Integrated flare prediction across IMIDs; digital biomarker panel development	PROPOSED	Validated in cardiology and neurology; translational research in IMIDs required

Evidence levels: ESTABLISHED Validated and in routine clinical use EMERGING Proof-of-concept in IMID context PROPOSED Theoretical rationale; prospective validation required

Supplementary Table S2. Fourier transform-based signal processing applications in clinical medicine, stratified by evidence level and potential relevance to immune-mediated inflammatory diseases. Part A documents established clinical applications in which FFT is standard practice; Part B describes emerging applications where proof-of-concept evidence exists in inflammatory disease contexts; Part C outlines proposed applications supported by theoretical rationale and requiring prospective validation. FFT, Fast Fourier Transform; IMID, immune-mediated inflammatory disease; RA, rheumatoid arthritis; SLE, systemic lupus erythematosus; SSc, systemic sclerosis; PsA, psoriatic arthritis; IBD, inflammatory bowel disease; LVV, large-vessel vasculitis; GCA, giant cell arteritis; TAK, Takayasu arteritis; NPSLE, neuropsychiatric SLE; ILD, interstitial lung disease; VW-MRI, vessel wall MRI; HRV, heart rate variability; LF/HF, low-frequency/high-frequency; PPG, photoplethysmography; PAH, pulmonary arterial hypertension; EMG, electromyography; FOT, forced oscillation technique; ABR, auditory brainstem response; ML, machine learning; CRP, C-reactive protein; ESR, erythrocyte sedimentation rate.
