# Supplementary material for: Artificial Intelligence and Machine Learning in Rheumatology and Systemic Inflammatory Diseases: From Pattern Recognition to Signal Analysis and Clinical Decision Support
Source: J Clin Med. 2026 Sep 4;15(17):6864. doi: 10.3390/jcm15176864 (PMC13566619; doi:10.3390/jcm15176864)
Supplement: Supplementary file 1 [file jcm-15-06864-s001.zip › Supplementary_Table_S3.pdf]

**Supplementary Table S3. Machine Learning Algorithms in Immune-Mediated Inflammatory Diseases: Clinical Task, Disease Context, and Reported Performance.**

| Algorithm / Model | Clinical Task | Disease / Dataset | Performance Metric | Reported Value | Notes |
|---|---|---|---|---|---|
| **SUPERVISED LEARNING — CLASSIFICATION AND PREDICTION** | | | | | |
| **Random Forest** | GPA vs MPA discrimination | AAV registry cohort | AUROC | **0.89** | Outperforms individual ANCA specificity; includes ENT, renal, pulmonary features |
| **Random Forest / Gradient Boosting** | Flare prediction | RA longitudinal cohort | AUROC | **0.75–0.88** | Key features: ACPA kinetics, CRP trend, power Doppler score |
| **Support Vector Machine (SVM)** | Flare prediction | SLE registry | AUROC | **0.82** | Integrates anti-dsDNA, complement, urinary biomarkers, PROs |
| **Logistic Regression + LASSO** | Anti-TNF response prediction | IBD pre-treatment cohort | AUROC | **0.80–0.88** | Mucosal gene expression + microbiome + serum proteomics |
| **CNN (ResNet/VGG)** | ISN/RPS lupus nephritis classification | Digitized renal biopsy slides | Accuracy vs pathologist | **≥Expert level** | Automated Berden/ISN-RPS class assignment |
| **CNN (U-Net)** | SSc-ILD extent quantification | HRCT dataset | Dice coefficient / ICC | **0.87–0.92** | Fibrotic vs inflammatory pattern differentiation |
| **CNN (real-time)** | Endoscopic disease activity (IBD) | Colonoscopy video frames | Sensitivity/Specificity | **≥90% / ≥85%** | Regulatory approved (FDA/CE mark) — clinical standard in IBD |
| **CNN (dermoscopy)** | PASI scoring (psoriasis) | Dermoscopic image dataset | Correlation vs dermatologist | **r > 0.95** | Dermatologist-level PASI estimation |
| **CNN (capillaroscopy)** | SSc pattern classification | Nailfold capillaroscopy images | Accuracy vs expert | **≥Expert level** | Early/active/late scleroderma pattern; reduces operator dependency |
| **UNSUPERVISED LEARNING — ENDOTYPING AND CLUSTER DISCOVERY** | | | | | |
| **k-means / Hierarchical clustering** | Molecular endotype discovery | SLE transcriptomic cohort | Silhouette score / Clinical validation | **Distinct endotypes validated** | IFN-high, complement-low, neutrophil-dominant subtypes with differential prognosis |
| **Non-negative Matrix Factorization (NMF)** | PR3-ANCA vs MPO-ANCA molecular distinction | AAV transcriptomic dataset | Supervised validation AUC | **0.85** | Molecular features beyond serological classification |
| **Consensus Clustering** | IBD molecular subtype discovery | Mucosal biopsy gene expression | Reproducibility across cohorts | **Validated in 3 independent cohorts** | Predicts anti-TNF response; microbiome features contribute independently |
| **Gaussian Mixture Model** | Myositis subgroup assignment | Autoantibody + clinical + histology | Agreement vs expert classification | **κ > 0.80** | Anti-MDA5, anti-TIF1γ, anti-Jo-1, anti-Mi-2 subgroups |
| **DEEP LEARNING — IMAGE ANALYSIS AND MULTI-MODAL INTEGRATION** | | | | | |
| **CNN (ResNet)** | Synovial histopathology classification | Digitized synovial biopsies | AUC vs pathologist | **0.87** | Lymphoid aggregates, lining layer hyperplasia, fibroblast activation |
| **CNN (YOLOv8 / Faster-RCNN)** | Halo sign detection (temporal artery US) | GCA ultrasound dataset | Sensitivity/Specificity | **88% / 91%** | Proof-of-concept; operator-independent consistency |
| **CNN (3D-UNet)** | PET/CT vascular FDG uptake quantification | LVV PET/CT dataset | AUROC (active vs inactive) | **0.89–0.94** | Surpasses visual expert scoring for LVV activity |
| **CNN + Radiomics** | Transmural inflammation (IBD) | MRI/CT enterography | AUROC vs endoscopy | **0.80–0.85** | Non-invasive alternative to colonoscopy in monitoring |
| **Transformer (ViT)** | Muscle MRI quantification (myositis) | Muscle MRI dataset | Correlation vs manual | **ICC > 0.90** | Edema and fatty replacement quantification |
| **NATURAL LANGUAGE PROCESSING — EHR MINING AND LLMS** | | | | | |
| **NLP (SVM / BERT)** | RA patient identification from EHR | >34 million clinical notes | AUROC | **0.98** | Outperforms ICD-code based identification |
| **NLP (BERT)** | Axial SpA identification from EHR | Primary care EHR dataset | Sensitivity/Specificity | **78% / 94%** | Enables earlier specialist referral from primary care |

| Algorithm / Model | Clinical Task | Disease / Dataset | Performance Metric | Reported Value | Notes |
|---|---|---|---|---|---|
| **NLP (outcome extraction)** | DAS28/SLEDAI from unstructured notes | Rheumatology EHR corpus | Sensitivity/PPV | **95% / 87%** | Enables large-scale retrospective disease activity reconstruction |
| **LLM (GPT-4 / Claude)** | Rheumatology board exam performance | Standardized MCQ datasets | Score vs rheumatologist | **At or above specialist level** | Hallucination risk highest in rare diseases (SAPHO, Still's, vasculitides) |
| **LLM + RAG (74 guidelines)** | Guideline-adherent clinical decision support | EULAR/ACR guideline corpus | Evaluator preference vs base LLM | **71–74%** | RAG reduces hallucination; investigational for clinical use |
| **REINFORCEMENT LEARNING AND SIGNAL PROCESSING** | | | | | |
| **Reinforcement Learning (DQN)** | Treat-to-target dose optimization | RA/SLE simulation cohort | Remission rate / GC exposure | **Superior to protocol-driven T2T** | Proof-of-concept; clinical validation ongoing |
| **FFT + SVM/Random Forest** | Wearable-based flare detection | RA/SLE accelerometry cohort | AUROC vs DAS28/SLEDAI | **0.78–0.85** | Frequency-domain features outperform raw time-domain inputs |
| **FFT + Spectral Entropy** | EMG myopathic pattern classification | Inflammatory myopathy EMG dataset | Sensitivity/Specificity vs manual | **85% / 88%** | Extension of established EMG spectral methodology |

**Supplementary Table S3.** Summary of machine learning and AI algorithms applied to immune-mediated inflammatory diseases, organized by algorithm class, clinical task, disease context, and reported performance metrics. Values are reported as published in the cited literature; comparative values represent head-to-head performance on the same dataset. AAV, ANCA-associated vasculitis; GPA, granulomatosis with polyangiitis; MPA, microscopic polyangiitis; RA, rheumatoid arthritis; SLE, systemic lupus erythematosus; IBD, inflammatory bowel disease; SSc, systemic sclerosis; GCA, giant cell arteritis; LVV, large-vessel vasculitis; SpA, spondyloarthritis; CNN, convolutional neural network; SVM, support vector machine; NLP, natural language processing; LLM, large language model; RAG, retrieval-augmented generation; FFT, Fast Fourier Transform; AUROC, area under the receiver operating characteristic curve; ICC, intraclass correlation coefficient; PPV, positive predictive value; PROs, patient-reported outcomes; HRCT, high-resolution CT; ILD, interstitial lung disease; PASI, Psoriasis Area and Severity Index; DQN, deep Q-network; T2T, treat-to-target; GC, glucocorticoids; DAS28, Disease Activity Score 28; SLEDAI, SLE Disease Activity Index; ENT, ear-nose-throat; MCQ, multiple choice questions.
