# Supplementary material for: Artificial Intelligence and Machine Learning in Rheumatology and Systemic Inflammatory Diseases: From Pattern Recognition to Signal Analysis and Clinical Decision Support
Source: J Clin Med. 2026 Sep 4;15(17):6864. doi: 10.3390/jcm15176864 (PMC13566619; doi:10.3390/jcm15176864)
Supplement: Supplementary file 1 [file jcm-15-06864-s001.zip › Supplementary_Table_S4.pdf]

Supplementary Table S4. Practical Guide for the Clinician: How to Critically Evaluate an AI Tool in Inflammatory Disease Medicine.

Question to Ask	Why It Matters	Red Flag	Caution	Reassuring
**1. DATA AND TRAINING**				
**What population was the model trained on?**	AI models reflect biases of their training data. A model trained on tertiary US/European centres may fail in your patient population.	RED FLAG<br>*Trained only on white, high-income, tertiary-centre patients; no demographic reporting*	CAUTION<br>*Some diversity reported but limited geographic/ethnic representation*	REASSURING<br>*Multicentre, multinational training cohort with demographic stratification reported*
**How large was the training dataset?**	Small datasets increase overfitting risk, especially for rare diseases. Performance on internal test sets overestimates real-world performance.	RED FLAG<br>*<500 patients for a classification task; rare IMID with <100 cases*	CAUTION<br>*500–2000 patients; single-centre validation only*	REASSURING<br>*>2000 patients; independent external validation cohort used*
**Were rare diseases included in training?**	LLMs and classifiers trained predominantly on common conditions hallucinate or underperform on rare IMIDs (SAPHO, Still's, vasculitides).	RED FLAG<br>*No mention of rare disease representation; LLM used without RAG for rare disease queries*	CAUTION<br>*Some rare disease data but underrepresented; RAG system available but not mandatory*	REASSURING<br>*Rare disease cases explicitly included; RAG anchored to specialty guidelines for rare IMIDs*
**2. VALIDATION AND EVIDENCE LEVEL**				
**Was the model validated externally?**	Internal test set validation systematically overestimates real-world performance. External validation is the minimum standard for clinical tools.	RED FLAG<br>*Validated only on a held-out portion of the same training dataset ("internal validation")*	CAUTION<br>*Single external validation cohort from a similar institution*	REASSURING<br>*Prospective validation in ≥2 independent, geographically distinct cohorts*
**What is the evidence level for this application?**	Most AI tools in IMIDs are proof-of-concept only. Regulatory clearance is the exception (IBD colonoscopy AI). Distinguish validated from investigational.	RED FLAG<br>*Proof-of-concept study presented as clinical tool; no peer-reviewed validation*	CAUTION<br>*Single prospective study; not yet replicated; performance metrics borderline*	REASSURING<br>*Regulatory clearance (FDA/EMA) OR multiple prospective validation studies with consistent AUC >0.80*
**Has it been tested in a prospective clinical setting?**	Retrospective studies with historical data do not capture real-world deployment challenges (data quality, operator variability, workflow integration).	RED FLAG<br>*Retrospective only; no prospective testing reported*	CAUTION<br>*Prospective feasibility study in single centre; no impact on clinical outcomes shown*	REASSURING<br>*Randomized or prospective multicenter study demonstrating clinical utility and/or improved patient outcomes*
**3. INTERPRETABILITY AND TRANSPARENCY**				
**Can the model explain its predictions?**	Black-box predictions without explanation are difficult to accept, challenge, or defend to patients. XAI methods make predictions auditable.	RED FLAG<br>*No XAI; pure black-box output with confidence score only*	CAUTION<br>*Post-hoc XAI available (SHAP/LIME) but not integrated into clinical interface*	REASSURING<br>*XAI explanations presented alongside predictions; clinician can review feature contributions*
**Are the features driving the prediction clinically plausible?**	Models can achieve high AUROC by learning spurious correlations (e.g., scanner brand, demographic proxies) rather than true pathological features.	RED FLAG<br>*Driving features are non-clinical (image artefacts, demographic variables, irrelevant metadata)*	CAUTION<br>*Driving features are clinical but partially unexpected; requires clinical interpretation*	REASSURING<br>*Driving features are clinically plausible, known disease biomarkers, and consistent with pathophysiology*
**Is the model a "black box" LLM for clinical decisions?**	LLMs generate fluent, confident-sounding outputs that may be factually incorrect (hallucination). Risk is highest for rare IMIDs.	RED FLAG<br>*Standalone LLM without RAG used for diagnosis/treatment in rare disease; no source citation*	CAUTION<br>*LLM with RAG but limited guideline coverage; sources not always cited*	REASSURING<br>*RAG system anchored to current EULAR/ACR guidelines; sources cited for every recommendation; human expert oversight maintained*
**4. REGULATORY AND IMPLEMENTATION**				
**Does the tool have regulatory clearance for clinical use?**	Medical AI without regulatory clearance is investigational. Using uncleared AI for clinical decisions carries medico-legal liability.	RED FLAG<br>*No regulatory clearance; marketed or used as "decision support" to bypass regulation*	CAUTION<br>*CE mark or FDA breakthrough device designation; clearance process ongoing*	REASSURING<br>*Full FDA 510(k)/De Novo clearance or CE marking for the specific intended use*

Question to Ask	Why It Matters	Red Flag	Caution	Reassuring
**Is the tool integrated into clinical workflow?**	Tools that require manual data export, separate login, or significant workflow disruption have low adoption rates regardless of performance.	RED FLAG<br>*Standalone application requiring manual data re-entry; not integrated with EHR*	CAUTION<br>*EHR integration available but requires significant IT configuration; partial workflow integration*	REASSURING<br>*Seamless EHR integration; alerts delivered at point of care; minimal workflow disruption*
**Who bears responsibility for AI-assisted decisions?**	AI tools are decision support, not decision makers. Clinical responsibility remains with the physician. Ensure your institution has an AI governance policy.	RED FLAG<br>*No clear governance policy; AI output treated as diagnostic/therapeutic decision*	CAUTION<br>*Institutional policy exists but unclear accountability for AI-assisted decisions*	REASSURING<br>*Clear governance framework; AI positioned as decision support; physician retains and documents final responsibility*
**5. ETHICAL AND EQUITY CONSIDERATIONS**				
**Could this tool worsen existing health disparities?**	If diagnostic delays disproportionately affect minority populations, AI trained on historical data may perpetuate or amplify these disparities.	RED FLAG<br>*No equity analysis; training data from historically underserved populations not included*	CAUTION<br>*Equity analysis mentioned but limited; subgroup performance not fully reported*	REASSURING<br>*Subgroup performance reported by race, ethnicity, sex, and socioeconomic status; performance gaps addressed in model development*
**Has the patient been informed of AI involvement?**	Patients have the right to know when AI tools contribute to their clinical care. Transparency is an ethical and increasingly legal requirement.	RED FLAG<br>*No patient disclosure; AI involvement not documented in clinical record*	CAUTION<br>*Institutional policy for disclosure exists; implementation inconsistent*	REASSURING<br>*Patient informed and consent documented; AI involvement noted in clinical record; patient can request human-only assessment*

Supplementary Table S4. A practical framework for the critical evaluation of artificial intelligence tools in the context of rheumatology and inflammatory disease medicine. For each key question, the table provides the clinical rationale and a three-level assessment scale: Red Flag (features that should deter clinical adoption without further validation), Caution (features that warrant careful evaluation before use), and Reassuring (features that support confidence in the tool's clinical utility and safety). This framework is intended to complement, not replace, institutional AI governance policies and regulatory guidance. AI, artificial intelligence; IMID, immune-mediated inflammatory disease; AUROC, area under the receiver operating characteristic curve; XAI, explainable AI; SHAP, SHapley Additive exPlanations; LIME, Local Interpretable Model-agnostic Explanations; LLM, large language model; RAG, retrieval-augmented generation; EHR, electronic health record; FDA, US Food and Drug Administration; CE, Conformité Européenne; EULAR, European Alliance of Associations for Rheumatology; ACR, American College of Rheumatology; SAPHO, synovitis, acne, pustulosis, hyperostosis, osteitis; IBD, inflammatory bowel disease; IT, information technology.
